# The Necessity of Regulating Drinking Scenes on Social Media Platforms Focusing on YouTube Sulbang Videos: Public Opinion From Surveys and YouTube Content Analysis

**DOI:** 10.2196/65162

**Published:** 2025-05-21

**Authors:** HyoRim Ju, HyeWon Lee, Juyoung Choi, EunKyo Kang

**Affiliations:** 1 Department of Family Medicine, Dankook University Hospital, Cheonan, Republic of Korea; 2 Department of Family Medicine, College of Medicine, Dankook University, Cheonan, Republic of Korea; 3 National Cancer Control Institute, National Cancer Center, 323 Ilsan-ro, Ilsandong-gu, Gyeonggi-do, Goyang, 10408, Republic of Korea, 82 1049004942; 4 National Cancer Center Graduate School of Cancer Science and Policy, National Cancer Center, Goyang, Republic of Korea; 5 Department of Family Medicine, National Cancer Center, Goyang, Republic of Korea

**Keywords:** alcohol, drinking scenes, social media, YouTube, sulbang, drinking scenes of social medial, public health, social media platforms, drinking regulate, alcohol policy

## Abstract

**Background:**

Alcohol consumption is a major risk factor for diseases and social burdens worldwide. Despite this, depictions of alcohol use continue to rise across various social media platforms, increasing concerns about their potential impact, particularly on adolescents. While some guidelines exist to regulate alcohol portrayals in media, they remain largely advisory and lack legal enforcement. As alcohol-related content becomes more widespread on social media, the need for stronger regulatory measures is growing.

**Objective:**

This study aimed to analyze the content of sulbang (broadcasts featuring alcohol consumption) on YouTube and to assess public opinions regarding the regulation of alcohol-related broadcasts on social media platforms such as YouTube.

**Methods:**

To evaluate public attitudes toward appropriate regulations on alcohol depictions in web-based media, a survey was conducted with 1500 adults (aged 20‐74 years) residing in South Korea. Participants were recruited through stratified multistage sampling, with a 21.8% (n=1500) response rate from 6880 invitations. The survey included Likert-scale and rank-ordered questions, with reliability assessed using Cronbach α. Additionally, a content analysis of 318 YouTube (sulbang) videos was conducted based on the Korean government’s media alcohol scene guidelines. Two trained coders independently analyzed the videos, achieving high intercoder reliability (Cohen κ=0.92).

**Results:**

This study found that exposure to sulbang content was significantly higher among individuals with higher education levels (n=33, 26.2% graduate degree holders), lower income groups (*P*<.001), and women. Younger individuals and heavy drinkers were also more likely to engage with such content, with heavy drinkers showing a significantly higher likelihood (*P*<.001). Regarding public opinion, 83.1% (n=1247) of respondents supported some form of regulation on sulbang content. However, heavy drinkers were less inclined to agree (coefficient: −0.3652; *P*<.001). Age was positively associated with stronger support for regulation (coefficient: 0.21984; *P*<.001), while women were significantly more likely than men to advocate for stricter restrictions (coefficient: 0.37827; *P*<.001). Exposure frequency also had the strongest correlation with support for regulation (coefficient: 1.0278; *P*<.001). The analysis of 318 YouTube videos revealed an average Like ratio of 97.9% (range: 32.7‐100.0), indicating predominantly positive viewer responses, with a median Video Power Index of 939.6 (range: 10.4-84,821.7). Content analysis based on the Media Drinking Scene Guidelines showed that 89.0% (n=283) of the videos glorified drinking, often portraying alcohol as a stress reliever or a source of recovery. Additionally, 92.8% (n=295) of the videos depicted binge drinking or drunkenness, and 27.7% (n=88) of the videos featured celebrities or notable figures consuming alcohol. Furthermore, 42.8% (n=136) of the videos presented distorted drinking norms, such as glorifying high tolerance or linking alcohol to sexual advances. In contrast, only 0.6% (n=2) of the videos were age-restricted, and 31.1% (n=99) included any warning message.

**Conclusions:**

Given the potential influence of alcohol-related content on drinking perceptions and behaviors, regulatory measures should be explored to mitigate possible risks. Strengthening content guidelines and increasing awareness could help address concerns about alcohol-related social media exposure.

## Introduction

Alcohol is a beverage made from ethanol and is one of the most widely used psychoactive substances worldwide. The World Health Organization classifies alcohol as a psychoactive substance due to its ability to alter brain function by affecting cognition, behavior, and consciousness [1]. This classification highlights the potential risks associated with alcohol consumption and underscores the need for public health intervention [2]. However, as of 2019, 43.8% of the global population aged 15 years and older consumed alcohol, with 38.0% of them engaging in heavy or binge drinking (defined as consuming at least 60 grams of alcohol on one or more occasions in the past month). Notably, about one in 5 adolescents aged 15‐19 years currently consume alcohol [3]. In South Korea, as of 2022, 58.7% of adults and 13.0% of adolescents reported drinking alcohol. This rate decreased during the COVID-19 pandemic but started to increase again in 2022 [4].

Alcohol use is a primary contributor to the global burden of disease and can lead to substantial health repercussions [5,6]. In particular, the link between alcohol and liver disease, such as liver cancer and cirrhosis is well-documented in numerous academic studies [7,8], while alcohol consumption is also associated with various nonliver cancers and heart conditions, including atrial fibrillation [9-11]. Moreover, alcohol use leads to societal problems such as drunk driving and violence, negatively affecting not only the drinkers themselves but also their families and society as a whole [12,13].

However, alcohol is often perceived as a social facilitator and stress reliever, associated with relaxation, social bonding, and celebration. This positive perception contributes to the normalization of alcohol use across various cultures [14]. Such perceptions are reinforced by media portrayals where celebrities are frequently shown consuming alcohol in television shows, movies, and advertisements. These depictions have the potential to normalize and glamorize alcohol consumption, influencing viewers’ attitudes and behaviors toward drinking [14-16]. Bandura’s Social Learning Theory provides a framework for understanding this phenomenon, suggesting that individuals, particularly adolescents, learn behaviors through observing others, especially when those behaviors are modeled by influential figures or portrayed positively in media. This theory underscores how repeated exposure to such portrayals can reinforce positive perceptions of drinking and normalize alcohol consumption [17,18]. Supporting this, previous research demonstrates that media exposure to drinking scenes is associated with the development of favorable attitudes toward alcohol and increased consumption among adolescents [18-20]. Accordingly, the role of media is emphasized in strategies aimed at reducing harmful levels of alcohol consumption, which include behaviors such as binge drinking, underage drinking, and excessive consumption that increase health risks and societal harm [2]. However, the majority of countries worldwide still do not have effective rules governing depictions of alcohol consumption in media and advertisements [3]. In 2017, South Korea introduced 10 guidelines aimed at minimizing the risks of media exposure to alcohol-related content. These guidelines sought to discourage positive portrayals of drinking and limit depictions of binge drinking. To address the evolving media landscape, including platforms like YouTube, the guidelines were revised and expanded to 12 in 2023 [21,22]. However, these guidelines remain advisory and lack legal enforcement. According to recent surveys by the Ministry of Health and Welfare and the Korea Health Promotion Institute, 66.3% of respondents reported watching alcohol-related scenes on TV or YouTube in the past year, with 27.6% being exposed to such scenes at least once a week [23]. Furthermore, the popularity of sulbang (alcohol-drinking broadcasts) on YouTube, a prominent social media platform, has increased exposure to alcohol-related content, which has become more provocative over time [24,25]. In addition, social media platforms like YouTube lack age restrictions for viewing alcohol-related broadcasts, allowing children and adolescents to easily access these sulbang videos, potentially influencing their positive perceptions of drinking [14-16]. Therefore, in the current context of increasing alcohol-related content on media platforms, there is a heightened need for the introduction of stronger regulatory policies for drinking-related content in media, including legal enforcement measures.

This study aims to analyze sulbang content on YouTube, one of the most prominent social media platforms, and to investigate public opinion on regulating alcohol-related broadcasts on social media. Additionally, it seeks to determine whether current (sulbang) content adheres to existing alcohol broadcasting guidelines and to provide evidence for the necessity of regulatory policies, such as age restrictions, on future alcohol-related videos. By addressing the gap between existing guidelines and the current media landscape, this study highlights the importance of implementing practical regulatory measures.

## Methods

### Study Design

Our study has two main components:

1. A cross-sectional survey was conducted among 1500 adults aged 20‐74 years residing in Korea to assess public attitudes toward appropriate levels of restrictions on drinking alcohol depictions in social media platforms.
2. To analyze the prevalence of “sulbang” on YouTube, a content analysis was performed on 318 YouTube videos with over 10,000 views identified through a search using the keyword “sulbang.”

### Participants and Procedures

We conducted a nationwide web-based survey targeting 1500 adults aged between 20 and 74 years. Our research sampling design was based on a stratified multistage sampling design using population data categorized by region, age, and sex provided by the National Statistical Office of Korea, thereby ensuring that survey participants represented the entire national population. Those who clicked on the link received an explanation about the study’s purpose.

Adequate explanations were provided to all participants, and informed consent was obtained before they participated in the survey. Among those who were invited, 21.8% of the participants consented to the research, resulting in a total of 1500 responses that were used for analysis.

### Data Collection and Questionnaire

The sociodemographic factors of survey participants collected in the study included age, gender, education level, household income, marital status, and residential area. Using a structured questionnaire, the participants’ frequency of alcohol consumption, the quantity consumed per drinking session, and the frequency of exposure to drinking alcohol scenes in social media, including press and web-based platforms, were assessed over the previous year. Additionally, to assess opinions on appropriate levels of restrictions on alcohol-related social media, we posed the following question: “Do you believe that alcohol scenes on social media platforms such as YouTube should be restricted? If you think it’s necessary, to what extent do you think it should be limited?” The response comprised the following options: (1) do not believe any restrictions on viewing are necessary; (2) limitations to a warning level cautioning about scenes featuring alcohol before viewing; (3) restriction for viewing only upon adult certification; (4) restrictions such as pixelation even with adult certification; and (5) restrictions prohibiting the inclusion of drinking alcohol scenes in videos.

### Sample Size and Data Analysis

Our target sample size was 1500 participants, which would allow for the description of responses with a 2.5% margin of error at a 95% confidence level. A descriptive analysis of the participants’ baseline characteristics as well as each item in the questionnaire was conducted, using frequencies and percentages. In addition, the study analyzed the relationship between sociodemographic factors and the frequency of exposure to scenes depicting alcohol consumption on social media platforms. It also conducted a regression analysis on perceptions of appropriate restriction levels for drinking scenes in social media and sociodemographic factors.

### YouTube Search Strategy and Data Collection

Using YouTube, the keywords “sulbang (alcohol-drinking shows),” “alcohol,” “drinking broadcast,” and “drinking” were searched. Results were sorted using YouTube’s default search option of “relevance-based ranking.” An analysis by an internet search engine revealed that over 90% of users typically select from the first 3 pages of search results, so 300 videos were chosen based on keywords collected through relevance-based rankings. A total of 318 videos were analyzed after excluding unrelated overlapping videos (eg, news) and videos with less than 10,000 views. Permission from YouTube was not required to conduct this study, as all the data used in our paper was publicly available, and no special access was needed for data collection.

### Extracted Variables

Data collected for each video included video ID, upload date, views, likes, dislikes, comments, and video length. Video Power Index was calculated as ([Likes * 100 / [Likes+Dislikes]] * [Views/Day] / 100). Video content analysis was evaluated based on the Media Drinking Scene Guidelines developed by the Ministry of Health and Welfare and the Korea Health Promotion Institute.

The guidelines were first developed in 2017 and revised in 2023. They include the 12 recommendations set out below.

1. Scenes involving drinking should only be included if absolutely necessary.
2. Positive portrayals of drinking should be avoided.
3. Illegal behavior or actions related to drinking alcohol that disrupt public order should not be portrayed as normal.
4. Videos must refrain from depicting risky behaviors such as violence, suicide, or sensational behavior related to drinking.
5. Videos should not depict teenagers drinking alcohol and should be extremely cautious about depicting teenagers together with adults consuming alcohol.
6. Scenes depicting celebrities or public figures consuming alcohol must be portrayed with caution in the video, considering their impact.
7. Videos should avoid depicting harmful drinking behaviors, such as binge drinking or drunkenness.
8. Drinking scenes should not be used as a means of advertising alcohol-related products.
9. Scenes that disregard the right to self-determination regarding drinking should be avoided.
10. A negative drinking culture should not be depicted as a typical situation.
11. Content that excessively highlights or glorifies drinking should be restricted to minimize access by children and adolescents through age restrictions, etc.
12. In scenes that excessively highlight or glorify drinking, the harmful effects of alcohol consumption must be communicated through warning signs.

The final YouTube video content coding was independently analyzed by 2 trained coders. The intercoder reliability was assessed using Cohen kappa (κ=0.92), yielding a value above 0.8, which indicates a high level of agreement. The coders underwent training sessions to ensure consistency and adherence to the coding protocol.

### Ethical Considerations

This study was approved by the National Cancer Center Ethics Board (project number: 2023-0284-0004). Before participation, all respondents were informed about the study’s purpose, procedures, potential risks, and benefits. Informed consent was obtained online before the survey began, and participation was entirely voluntary. No compensation was provided to participants. Access to the data was restricted to authorized researchers only. To ensure privacy and confidentiality, all collected data were fully anonymized and deidentified before analysis.

## Results

### Sociodemographic Factors and the Frequency of Exposure to Alcohol-Drinking Videos

Table 1 shows the relationship between sociodemographic variables and the frequency of exposure to depictions of alcohol consumption on social media platforms. Although no statistically significant variances based on gender were observed, a higher percentage of women than men reported viewing social media content depicting alcohol consumption more than 4 times a week. There were no significant differences in age or marital status, but the younger age groups tended to view more social media containing alcohol consumption. Conversely, a significant difference was observed for educational level. Specifically, the group with higher education levels had the highest proportion of individuals viewing alcohol-related content more than 4 times a week (n=60, 16.9% for up to high school graduates; n=194, 19.0% for college graduates; and n=33, 26.2% for those with graduate degrees or higher; *P*<.001).

**Table 1.** The relationship between sociodemographic factors and the frequency of exposure to scenes depicting alcohol consumption in social media including web-based platforms: analysis of a nationwide survey of South Korean adults (N=1500).

	Total (N=1500), n (%)	The frequency of exposure to scenes depicting alcohol consumption in social media						*P* value
		None (n=220), n (%)	≤1 time/moment (n=205), n (%)	≤1 time/week (n=327), n (%)	2‐3 times/week (n=461), n (%)	4‐6 times/week (n=209), n (%)	Every day (n=78), n (%)	
Gender								.11
Men	761 (50.7)	111 (14.6)	102 (13.4)	188 (24.7)	228 (30.0)	95 (12.5)	37 (4.9)	
Women	739 (49.3)	109 (14.8)	103 (13.9)	139 (18.8)	233 (31.5)	114 (15.4)	41 (5.6)	
Age (years)								.07
20‐29	242 (16.1)	30 (12.4)	26 (10.7)	53 (21.9)	71 (29.3)	47 (19.4)	15 (6.2)	
30‐39	246 (16.4)	35 (14.2)	40 (16.3)	42 (17.1)	78 (31.7)	39 (15.9)	12 (4.9)	
40‐49	307 (20.5)	42 (13.7)	34 (11.1)	67 (21.8)	107 (34.9)	38 (12.4)	19 (6.2)	
50‐59	331 (22.1)	43 (13.0)	45 (13.6)	82 (24.8)	105 (31.7)	37 (11.2)	19 (5.7)	
60‐69	288 (19.2)	53 (18.4)	50 (17.4)	68 (23.6)	73 (25.4)	34 (11.8)	10 (3.5)	
70+	86 (5.7)	17 (19.8)	10 (11.6)	15 (17.4)	27 (31.4)	14 (16.3)	3 (3.5)	
Marital status								.10
Single	538 (35.9)	81 (15.1)	74 (13.8)	114 (21.2)	152 (28.3)	84 (15.6)	33 (6.1)	
Married	888 (59.2)	121 (13.6)	118 (13.3)	201 (22.6)	293 (33.0)	113 (12.7)	42 (4.7)	
Separated, divorced, or widowed	74 (4.9)	18 (24.3)	13 (17.6)	12 (16.2)	16 (21.6)	12 (16.2)	3 (4.1)	
Educational status								<.001
≤ High school	355 (23.7)	77 (21.7)	57 (16.1)	71 (20.0)	90 (25.4)	44 (12.4)	16 (4.5)	
Bachelor’s degree	1,019 (67.9)	127 (12.5)	134 (13.2)	230 (22.6)	334 (32.8)	143 (14.0)	51 (5.0)	
≥ Graduate degree	126 (8.4)	16 (12.7)	14 (11.1)	26 (20.6)	37 (29.4)	22 (17.5)	11 (8.7)	
Household income (US $)								<.001
<2000	288 (19.2)	68 (23.6)	42 (14.6)	59 (20.5)	58 (20.1)	44 (15.3)	17 (5.9)	
2000‐3999	628 (41.9)	81 (12.9)	101 (16.1)	129 (20.5)	198 (31.5)	88 (14.0)	31 (4.9)	
4000‐5999	310 (20.7)	29 (9.4)	35 (11.3)	76 (24.5)	109 (35.2)	49 (15.8)	12 (3.9)	
≥6000	274 (18.3)	42 (15.3)	27 (9.9)	63 (23.0)	96 (35.0)	28 (10.2)	18 (6.6)	
Self-reported health status								.53
Excellent to very good	486 (32.4)	62 (12.8)	73 (15.0)	110 (22.6)	156 (32.1)	57 (11.7)	28 (5.8)	
Good	827 (55.1)	126 (15.2)	112 (13.5)	182 (22.0)	246 (29.7)	121 (14.6)	40 (4.8)	
Fair to poor	187 (12.5)	32 (17.1)	20 (10.7)	35 (18.7)	59 (31.6)	31 (16.6)	10 (5.3)	
Smoking								<.001
Never-smoker	458 (30.5)	45 (9.8)	38 (8.3)	103 (22.5)	171 (37.3)	73 (15.9)	28 (6.1)	
Current smoker	282 (18.8)	29 (10.3)	44 (15.6)	73 (25.9)	89 (31.6)	35 (12.4)	12 (4.3)	
Ex-smoker	760 (50.7)	146 (19.2)	123 (16.2)	151 (19.9)	201 (26.4)	101 (13.3)	38 (5.0)	
Drinking alcohols								<.001
Nondrinking (Abstainer)	272 (18.1)	90 (33.1)	41 (15.1)	45 (16.5)	49 (18.0)	33 (12.1)	14 (5.1)	
Social drinker	660 (44.0)	87 (13.2)	95 (14.4)	146 (22.1)	214 (32.4)	92 (13.9)	26 (3.9)	
Heavy drinker	568 (37.9)	43 (7.6)	69 (12.1)	136 (23.9)	198 (34.9)	84 (14.8)	38 (6.7)	

Significant differences in exposure to alcohol-related content in media were observed based on income levels. An analysis focusing on the frequency of individuals who viewed social media featuring alcohol consumption more than 4 times a week indicated that the lowest income group ( US $2000 per month) exhibited a higher tendency to see content associated with drinking (*P*<.001). The study did not find a significant association between self-reported health status and exposure to alcohol-related content on social media; however, a significant correlation was observed between smoking and alcohol consumption. Specifically, heavy drinkers were significantly more likely to view alcohol-related content on social media platforms (*P*<.001).

### Opinion on Regulating Scenes Depicting Alcohol Consumption in Social Media Platforms

**Figure 1.**
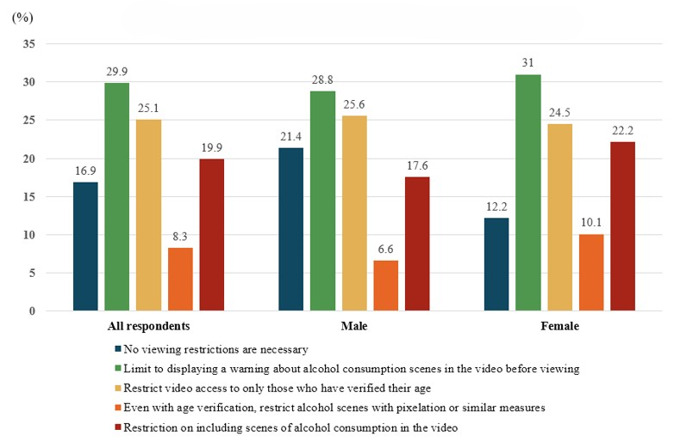
Opinion on regulating scenes depicting alcohol consumption in social media platforms: Analysis of a nationwide survey of South Korean adults (N=1500).

Figure 1 illustrates the opinions regarding the regulation of scenes depicting alcohol consumption on social media platforms, including web-based platforms such as YouTube. Among the respondents, 16.9% (n=253) said that viewing restrictions were unnecessary, while 29.9% (n=449) said that they believed cautionary warnings about alcohol-related scenes before viewing were essential. Additionally, a significant proportion (376/1500, 25.1%) felt that restrictions requiring adult certification for viewing were necessary, while 8.3% (n=124) of respondents advocated for stricter measures, such as pixelation, even with adult certification. Finally, 19.9% (n=298) of respondents supported restrictions that would prohibit the inclusion of alcohol consumption scenes in videos. Notably, women expressed a stronger need for restrictions compared with men, with 22.2% (n=164) of women believing that restrictions on drinking videos were necessary, compared with 17.6% (n=134) of men. The data also revealed that 21.4% (n=163) of men and 12.2% (n=90) of women believed that there should be no restrictions on drinking videos, further indicating that women were more in favor of restrictions on such videos.

### Regression Analysis of Perceptions of the Appropriate Level of Restriction for Drinking Scenes

Table 2 shows the results of an analysis conducted on the factors influencing perceptions of restrictions on drinking scenes. The analysis considered demographic factors such as age and gender, as well as the association between the amount of alcohol consumed and the level of exposure to drinking scenes on social media with perceptions of restrictions on drinking scenes. The perception of the level of restrictions on drinking scenes in social media was found to be positively associated with age, with a coefficient of 0.21984, and a *P* value of <.001. Accordingly, the analysis confirmed that older age groups believe that scenes depicting drinking on social media should have greater restrictions.

Regarding gender differences, women were significantly more likely than men to advocate for stricter limitations on depictions of drinking on social media (coefficient: 0.37827; *P*<.001), although no significant variances were observed for education level, marital status, household income, or self-reported health status.

**Table 2.** Regression analysis of perceptions of the appropriate level of restriction for drinking scenes in web-based social media and sociodemographic factors: analysis of a nationwide survey of South Korean adults (N=1500).

Variable	Coefficient	Standard error	*Z* value	*P* value
Age	0.21984	0.03993	5.505	<.001
Gender	0.37827	0.09352	4.045	<.001
Educational status	0.03793	0.08976	0.423	.67
Marital status	−0.0661	0.10786	−0.613	.54
Household Income	−0.02224	0.0493	−0.451	.65
Self-reported health status	0.02753	0.06323	0.435	.66
Drinking amount of alcohols	−0.3652	0.06605	−5.53	<.001
Exposure to scenes depicting alcohol consumption in web-based social media	1.0278	0.16492	6.23	<.001

However, in relation to the quantity of alcohol consumed, there was a stronger tendency to support less stringent restrictions on drinking scenes as alcohol consumption increased (coefficient: −0.3652; *P*<.001). The exposure level to drinking scenes showed the strongest correlation (coefficient: 1.0278; *P*<.001), with individuals who were more frequently exposed to drinking scenes tending to hold stronger opinions regarding the necessity for increased restrictions on these scenes.

### Descriptive Features and Video Content Analysis of “Sulbang”

The analysis encompassed 318 YouTube videos, with a Like ratio of 97.9% (range: 32.7‐100.0), indicating a prevalent positive reception among viewers. The median Video Power Index value stood at 939.6 (ranging from 10.4 to 84,821.7; Table 3). Examination of alcohol-related content within drinking scenes, in accordance with the Media Drinking Scene Guidelines, revealed that approximately 89.0% (n=283) of the content glorified drinking (Table 4). Videos showing positive portrayals often suggest that alcohol helps with stress relief and can rejuvenate a fatigued body after just one drink. Videos featuring celebrities or notable figures consuming alcohol constituted 27.7% (n=88) of the total, while 92.8% (n=295) depicted either binge drinking or drunkenness. Videos that undermined the autonomy of others and promoted drinking accounted for 15.7% (n=50) of the total, with 42.8% (n=136) depicting a distorted drinking culture, often emphasizing high alcohol tolerance or portraying alcohol as a catalyst for sexual advances. Conversely, just 0.6% (n=2) of the videos were age-restricted to viewers younger than 19 years, and only 31.1% (n=98) included warnings.

**Table 3.** Characteristics of YouTube (sulbang) videos related to alcohol consumption (n=318).

Variable	Median (range)
Views	298,556 (10,404-17,790,441)
Video length (minutes)	14.8 (0.9‐153.1)
Time on YouTube (days)	362.0 (126-2,505)
Comments	450.5 (8-23,845)
Likes (thumbs up)	5481 (10-832,067)
Dislikes (thumbs down)	150 (0‐13,875)
Like ratio	97.9 (32.7‐100.0)
View ratio	1030.5 (10.5‐85,944.2)
Video power index	939.6 (10.4‐84,821.7)

**Table 4.** Content analysis of YouTube (Sulbang) videos based on the 2023 Korean media drinking scene guidelines (n=318).

Media drinking scene guidelines	Not following the guidelines, n (%)
1. Scenes involving drinking should be minimized and only included if absolutely necessary.	3 (0.9)
2. Positive portrayals of drinking should be avoided.	283 (89.0)
3. Illegal behavior or actions related to drinking alcohol that disrupt public order should not be portrayed as normal.	5 (1.6)
4. Videos must refrain from depicting risky behaviors such as violence, suicide, or sensational behavior related to drinking.	4 (1.3)
5. Videos should not depict teenagers drinking alcohol and should be very careful about depicting teenagers together with adults consuming alcohol.	0 (0.0)
6. Scenes depicting celebrities or public figures consuming alcohol must be portrayed with caution in the video, considering their impact.	88 (27.7)
7. Videos should avoid depicting harmful drinking behaviors, such as binge drinking or drunkenness.	295 (92.8)
8. Drinking scenes should not be used as a means of advertising alcohol products.	37 (11.6)
9. Scenes that disregard the right to self-determination regarding drinking should be avoided.	50 (15.7)
10. A negative drinking culture should not be depicted as a typical situation.	136 (42.8)
11. Contents that excessively highlights or glorifies drinking should be restricted to minimize access by children and adolescents through age restrictions, etc	2 (0.6)
12. In scenes that excessively highlight or admire drinking, the harmful effects of alcohol consumption must be communicated through warning signs	98 (31.1)

## Discussion

### Principal Findings

Alcohol consumption is a major global health risk factor and social burden [26,27], yet it has been positively perceived as a means of stress relief and fostering social bonds in everyday life [14]. This perception has normalized alcohol use across various cultures, and exposure to drinking scenes has increased, particularly due to the rise in social media platform usage, such as YouTube, following the COVID-19 pandemic [28,29]. In South Korea, based on previous studies that suggested repeated exposure to drinking scenes could lead to the normalization of alcohol use and the glorification of its effects, 10 media drinking scene guidelines were introduced in 2017 to regulate exposure to drinking scenes in media. This effort was further enhanced in 2023 with updates that included age restrictions and warning labels, especially considering the pronounced impact on adolescents and young adults [21,22]. However, these guidelines remain legally unenforceable.

This study aims to emphasize the importance of implementing practical regulatory measures for social media platforms by analyzing sulbang (alcohol-themed broadcasting) content on YouTube and examining public opinion on regulating alcohol-related broadcasts on social media. The study explores the correlations between sociodemographic factors, frequency of exposure to drinking scenes on YouTube, and the global scope of regulations on such scenes. It also evaluates whether the increased sulbang content on YouTube aligns with the media drinking scene guidelines. The findings reveal that women, individuals with higher education levels, or those with lower incomes were more frequently exposed to drinking content. Although there was no significant difference between age groups, younger individuals tended to watch more alcohol-related social media content. Additionally, individuals with higher alcohol consumption reported greater engagement in alcohol-related content on social media platforms. Regarding opinions on regulating YouTube drinking content, women and older adults showed a stronger tendency to support stricter regulations. There was also a positive correlation between exposure to drinking scenes and belief in the necessity of regulation (greater exposure was associated with stronger support for regulation) and a negative correlation between alcohol consumption and belief in the necessity of regulation (higher consumption was associated with lower support for regulation). This may suggest that heavy drinkers perceive drinking as more normalized due to repeated exposure, which aligns with Bandura’s social learning theory, explaining how observed behaviors can shape perceptions and habits [17,18]. Finally, an evaluation of YouTube (sulbang) content based on the media drinking scene guidelines showed that many videos portrayed alcohol consumption positively or emphasized binge drinking and distorted drinking cultures. However, relatively few videos included age restrictions or warning labels. These findings demonstrate a lack of enforcement mechanisms in web-based media regulations and highlight the need for future legally binding regulations.

Previous studies have widely demonstrated that exposure to drinking scenes in media increases positive perceptions of alcohol consumption and drinking behavior [30-32]. This study aligns with those findings, particularly emphasizing the tendency of binge drinkers to watch alcohol-related content more frequently and to have lower awareness of the need for regulation. This suggests that repeated exposure may reinforce positive attitudes toward drinking and lead to resistance against regulation [33]. According to 2021 high-risk drinking statistics in South Korea, while the high-risk drinking rate among men has decreased, it has increased among women, particularly among men aged 40-50 years and women aged 20-30 years [34]. This supports previous findings that women are more emotionally influenced by drinking scenes in media [35,36]. These results underline the need for tailored regulatory and educational strategies targeting specific demographic groups.

Currently, most countries enforce offline alcohol regulations, such as taxation and age restrictions on sales, but regulations for alcohol-related content on social media remain nonbinding recommendations. Considering the negative impacts of exposure to drinking scenes and the necessity of regulation, strong legal measures, such as age restrictions, broadcasting time regulations, and warning messages before broadcasts, should be swiftly implemented to effectively control the exposure to alcohol content on social media.

### Limitations

This study has several limitations. First, the analysis focused solely on YouTube as the research platform. However, YouTube is the most widely used video-sharing platform worldwide and records the highest viewership among social media platforms. Moreover, it provides videos of sufficient length to analyze drinking behaviors. Therefore, analyzing only YouTube allowed for the collection of a sufficiently large data set. Second, there is no validated psychometric tool specifically designed to assess exposure to alcohol-related content and regulatory attitudes. As a result, a separate questionnaire was designed for this study. Although the survey instrument was not a previously validated tool, considerable effort was made to ensure the accuracy and reliability of the study results. To improve representativeness, a stratified sampling method was adopted based on region, age, and gender. Additionally, structured response options and clear definitions of key concepts were provided to minimize ambiguity and enhance response reliability. Another limitation is that this study indirectly assessed exposure to drinking scenes through a survey rather than directly measuring actual exposure. To address this, the survey items related to alcohol consumption were carefully structured to be as specific as possible. Finally, since this study is a cross-sectional study, it has limitations in explaining causal relationships. These limitations have been explicitly acknowledged in the conclusion, and future research should use data analytics and experimental methodologies to achieve more objective and direct evaluations.

### Conclusions

This study aimed to analyze sulbang (alcohol-themed broadcasting) content on YouTube and investigate public opinion regarding the regulation of alcohol-related broadcasts on social media platforms like YouTube. The findings indicated that individuals with higher tendencies toward alcohol consumption were more likely to frequently watch sulbang content and generally opposed regulatory measures. However, the majority of survey participants agreed on the necessity of regulating (sulbang) content. Additionally, an evaluation of sulbang content based on media drinking scene guidelines showed that most videos portrayed alcohol consumption positively or included binge drinking, while very few videos featured age restrictions or warning labels. Considering the potential influence of such content in shaping attitudes toward alcohol consumption and the possible impacts of increased exposure, it is essential to consider regulatory approaches for alcohol-related content on social media.

This study highlights the potential association between positively portrayed alcohol consumption content and public attitudes but cannot confirm causal relationships due to its reliance on cross-sectional data. To address this limitation, further research using longitudinal or experimental methodologies is needed. Such studies would play a crucial role in assessing the long-term effects of exposure and evaluating the effectiveness of proposed regulatory measures.
